# Hemoptysis in Cancer Patients

**DOI:** 10.3390/cancers15194765

**Published:** 2023-09-28

**Authors:** Emad D. Singer, Saadia A. Faiz, Aiham Qdaisat, Karim Abdeldaem, Jim Dagher, Patrick Chaftari, Sai-Ching J. Yeung

**Affiliations:** 1 Department of Emergency Medicine, The University of Texas MD Anderson Cancer Center, Houston, TX 77030, USA; edsinger@mdanderson.org (E.D.S.); aqdaisat@mdanderson.org (A.Q.); ksabdeldaem@mdanderson.org (K.A.); 2 Department of Abdominal Imaging, Division of Diagnostic Imaging, The University of Texas MD Anderson Cancer Center, Houston, TX 77030, USA; 3 Department of Pulmonary Medicine, The University of Texas MD Anderson Cancer Center, Houston, TX 77030, USA; 4 Faculty of Medicine, Saint Joseph University of Beirut, Beirut 1100, Lebanon

**Keywords:** hemoptysis, cancer, prediction score, lung cancer, etiology, risk factors, investigations, management, prognosis, oncology

## Abstract

**Simple Summary:**

This review explores hemoptysis in cancer patients. It delves into risk factors, epidemiology and clinical consequences. The need for a mortality prediction score for cancer patients with hemoptysis-related variables is needed, and this tool could aid in risk assessment, optimize the control of bleeding with critical care, implement tracheobronchial or vascular interventions, and guide treatment planning. Managing hemoptysis in cancer patients requires addressing the underlying cause and providing supportive care for improved quality of life.

**Abstract:**

Hemoptysis in cancer patients can occur for various reasons, including infections, tumors, blood vessel abnormalities and inflammatory conditions. The degree of hemoptysis is commonly classified according to the quantity of blood expelled. However, volume-based definitions may not accurately reflect the clinical impact of bleeding. This review explores a more comprehensive approach to evaluating hemoptysis by considering its risk factors, epidemiology and clinical consequences. In particular, this review provides insight into the risk factors, identifies mortality rates associated with hemoptysis in cancer patients and highlights the need for developing a mortality prediction score specific for cancer patients. The use of hemoptysis-related variables may help stratify patients into risk categories; optimize the control of bleeding with critical care; implement the use of tracheobronchial or vascular interventions; and aid in treatment planning. Effective management of hemoptysis in cancer patients must address the underlying cause while also providing supportive care to improve patients’ quality of life.

## 1. Definition

Hemoptysis refers to the expectoration of blood, alone or mixed with mucus, from the lower respiratory system (i.e., below the vocal cords), and it engenders significant concern in the both the patient and healthcare team. Often, hemoptysis is minimal and related to an inflammatory or infectious etiology, and it typically entails less than 100 mL over 24 h [1,2,3]. Although massive hemoptysis is rare, it may account for 5% to 15% of episodes and should be treated as a life-threatening condition necessitating effective assessment and management [4]. Defining massive hemoptysis quantitatively has been a challenge and a source of debate in the literature. Prior definitions of massive hemoptysis range widely from greater than 200 mL to greater than 1000 mL of blood within 24 h [3,5,6,7]; further, the amount of blood reported by patients and their families is often under- or overestimated. Also, blood from the lower respiratory tract must be differentiated from that of the upper respiratory tract and/or the upper gastrointestinal tract. Moreover, clinical factors are often much more important in terms of outcomes and include the briskness of bleeding, the patient’s ability to maintain a patent airway and expectorate blood, the availability of specialized support for therapeutic options, and the patient’s underlying physiological reserve. With this in mind, major or massive hemoptysis can more practically be defined as hemoptysis that causes severe consequences resulting in aspiration of blood to the contralateral lung, airway obstruction, hypoxemia and respiratory failure requiring additional respiratory support, including mechanical ventilation, severe anemia necessitating transfusion and, in extreme cases, even death [8,9].

## 2. Pathophysiology

The lung receives blood supply from two distinct sources: pulmonary arteries, which flow to the alveoli for gas exchange and are a low-pressure circulation, and bronchial arteries, which supply oxygenated blood to the bronchi and visceral pleura and are a high-pressure circulation [10,11]. Although the bronchial arteries make a smaller contribution to pulmonary blood flow than the pulmonary arteries, they more frequently give rise to hemoptysis, especially when they undergo neovascularization in the presence of inflammatory diseases. These newly developed bronchial arteries are ensconced within smooth muscle fibers that can contract in response to diverse stimuli, increasing the risk of bleeding. Effective elimination of this neovascular vessel network can be achieved through arterial embolization. On the other hand, vasospasms in the pulmonary arterial network are comparatively weak, as these vessels possess thin and delicate walls that lack active contractile properties [10,11]. Pulmonary arterial hemorrhage typically arises from ulceration of the vessel wall, which may be due to lung cancer or other diseases. In such cases, bleeding is often transiently controlled by clot formation. However, the risk of rebleeding remains if the clot dissolves or the vessel wall ruptures again [10,11]. Unfortunately, it is not always possible to determine the arterial origin of the hemorrhage.

In the context of cancer, hemoptysis may occur through various pathophysiological mechanisms, impacting the amount and speed of the bleeding [12,13]. These include the formation of angiogenesis in and around the tumor, shedding of the tumor’s outer layer, necrosis within the tumor, irritation caused by coughing, erosion of airway tissue into nearby blood vessels and bleeding resulting from medical procedures or treatments [12,13]. Certain therapies targeting vascular endothelial growth factor (VEGF), such as bevacizumab, may also result in hemoptysis due to the distribution of endothelial cell regulation reversing the protective effect of VEGF on endothelial cells [14,15], as well as fistulous tracts when such agents are combined with radiation [16,17]. Other medications, including anticoagulants and antiplatelet therapies that are commonly used in cancer patients, can also cause or exacerbate bleeding.

## 3. Etiology

**Figure 1:**
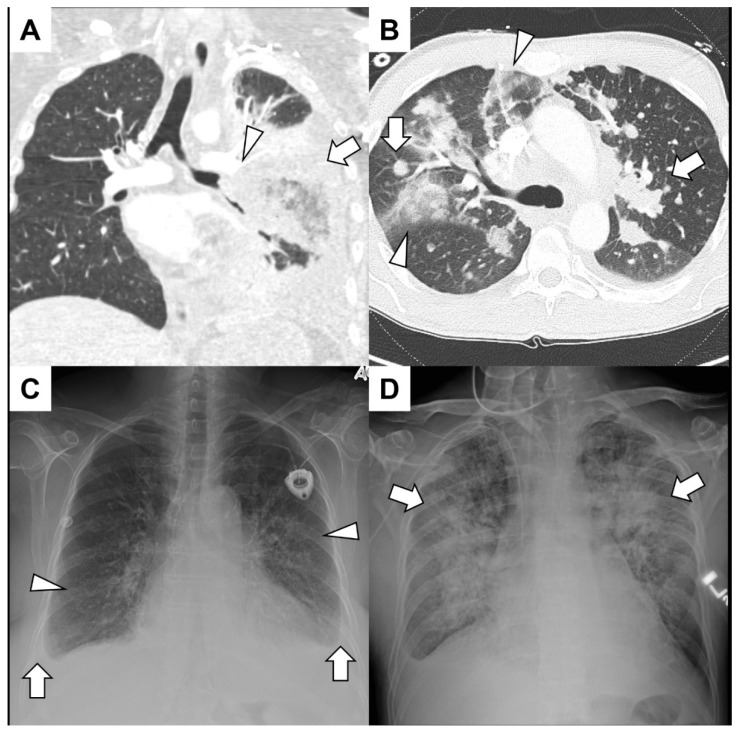
(**A**) A young man with acute lymphoid leukemia presented with significant hemoptysis. He had both neutropenia and lymphopenia due to recent treatments. A CT angiogram showed irregular soft-tissue encasement of the lower-order branches of the left pulmonary artery (arrowhead) and consolidation (arrow) and air bronchograms in the left lower lobe. Bronchoscopy revealed that the left lower lobe collapsed without endobronchial lesions. Surgical intervention with left lower lobectomy confirmed invasive mucormycosis and left pulmonary artery branches were full of thrombi. (**B**) A middle-aged man with adenoid cystic cancer presented with hemoptysis mixed with mucus over the previous few weeks. A CT angiogram excluded pulmonary emboli but showed diffuse nodules and mass-like consolidation (arrows) along with ground glass infiltrates peripherally (arrowhead). No endobronchial component was identified, and since the hemoptysis was of a small volume, no further intervention was warranted. (**C**) An elderly woman with a history of Hodgkin lymphoma presented with hemoptysis, dyspnea and a syncopal episode. A chest radiograph demonstrated bilateral infiltrates (arrowheads) and small bilateral effusion (arrows) with blunting of the costophrenic angles. She had volume overload with pulmonary edema, and an echocardiogram demonstrated severe aortic valve stenosis. (**D**) A patient with acute myelogenous leukemia after hematopoietic allogenic stem cell transplantation from a matched sibling presented with hemoptysis and epistaxis. A chest radiograph showed bilateral diffuse infiltrates (arrows), and a bronchoscopy revealed blood-tinged secretions throughout the tracheobronchial tree. He had diffuse alveolar hemorrhage due to thrombotic microangiopathy related to calcineurin inhibitors.

**Figure 2:**
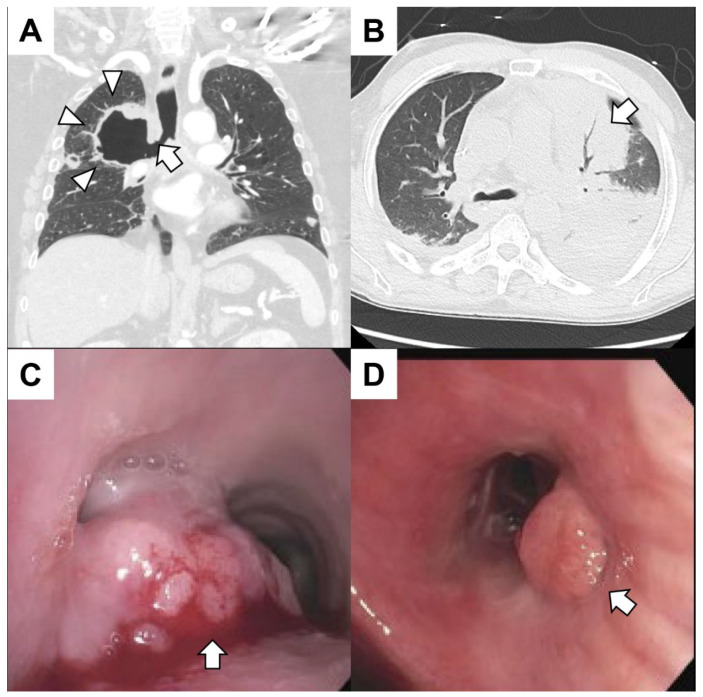
(**A**) A patient with metastatic lung cancer treated with radiation 1 month prior to the right upper lobe presented with hemoptysis. The coronal CT of the chest revealed a cavitary lesion (arrowheads) contiguous with the right mainstem bronchus (arrow). The patient expired from massive hemoptysis shortly after presenting to the emergency center. (**B**) A patient with leukemia with pancytopenia (white blood cells 0.0 × 10^9^/L platelets 14,000 × 10^9^/L) and fever presented with hemoptysis. The patient had sepsis with bacteremia. A chest CT revealed consolidation in the left upper lobe (arrow) with air bronchograms. (**C**) A patient with gastroesophageal junction cancer presented with dyspnea and mild hemoptysis. Bronchoscopy revealed a mass at the main carina obstructing 90% of the left mainstem and 20% of the right mainstem. The mass (arrow) was large, exophytic and friable. The tumor was debulked using rigid bronchoscopy, and argon plasma coagulation was used to achieve hemostasis. Biopsies were consistent with metastatic disease. (**D**) A patient with metastatic colorectal cancer had dyspnea and mild hemoptysis. The patient had mucopurulent and blood-tinged secretions emanating from the right middle lobe. An endoluminal tumor mass (arrow) obstructing the superior segment of the left lower lobe was found. The hemoptysis resolved after treatment of a respiratory infection.

Hemoptysis can arise from a diverse range of etiologies, including lung cavitation, infections, neoplastic conditions, pulmonary thromboembolic disease, cardiac dysfunction, including pulmonary edema or valvular abnormalities, hematological disorders, iatrogenic factors, sequelae from drugs or other substances, vascular anomalies, congenital lung malformations and idiopathic origins [18,19,20,21,22,23]. Examples of patients presented with hemoptysis in the setting of cancer are shown in Figure 1 and Figure 2.

Immune checkpoint inhibitor therapy is currently the standard of care for many types of malignancies and has dramatically changed the landscape for treating patients with cancer. Among the various immune-related adverse events (irAEs) associated with immune checkpoint inhibitor therapy, diffuse alveolar hemorrhage (DAH) is a rare but a concerning and life-threatening side effect [24,25,26,27]. Also, certain antineoplastic therapies, severe thrombocytopenia, and post-hematopoietic allogeneic stem cell transplantation can cause hemoptysis in cancer patients. Characterized by dyspnea, cough and hemoptysis, DAH often necessitates bronchoscopic evaluation to guide diagnosis and differentiate cases with infectious, bland hemorrhage and connective tissue disease–associated causes from those induced by cytotoxic drugs and graft-versus-host disease [28]. The prognosis of DAH is poor, with in-hospital mortality ranging from 20% to 100%. Identifying the underlying etiology of hemoptysis is paramount to ensure timely and targeted therapeutic interventions and provide the necessary psychosocial support [18,19,20,21]. Table 1 summarizes the common etiologies of hemoptysis and the causes of life-threatening hemoptysis [21,29,30,31].

## 4. Epidemiology of Hemoptysis

The epidemiology of hemoptysis varies widely based on geographic location, epidemiological study design and definitions of hemoptysis. Minor hemoptysis commonly results in outpatient clinic visits and hospital admissions, and hemoptysis comprises 10% to 15% of all pulmonary consultations [49]. Interestingly, in a recent observational, prospective multicenter study in Italy, malignancy, bronchiectasis and pneumonia were the main etiologies of hemoptysis; pneumonia and acute bronchitis resulted in mild hemoptysis, and neoplasms and bronchiectasis resulted in moderate to severe hemoptysis [49]. Massive hemoptysis is relatively infrequent, and the leading causes of massive hemoptysis worldwide include tuberculosis, bronchiectasis, mycetoma and cancer [8,49]. In the United States, hemoptysis is the reason for approximately 6.8% of visits to pulmonary clinics and is responsible for about 11% of admissions to pulmonary hospital services [8].

Approximately 20% of hemoptysis cases are caused by lung cancer, with 3% of lung cancer cases resulting in massive hemoptysis [12]. The highest incidence of bleeding is linked to squamous cell histology, and massive hemoptysis is linked to cavitation and tumors originating from or penetrating through the central airways [50,51]. According to Corey and associates, individuals with lung cancer and hemoptysis exhibited a mortality rate of 59%. However, this figure escalated to 80% for those who experienced bleeding exceeding 1000 mL within a 24 h period in conjunction with a cancer diagnosis [52]. In addition, inpatient hospitalization, advanced age and a requirement for mechanical ventilation are independent predictors of in-hospital mortality among patients with nonsmall cell lung cancer [53]. Other mortality predictors include contralateral lung aspiration on radiography and hemodynamic instability [29]. Other tumors including thyroid, breast, colon and renal cancer, as well as melanoma, may also infiltrate the tracheobronchial tree, resulting in malignant airway obstruction and hemoptysis [54].

## 5. Presentations and Investigations for Hemoptysis in Cancer Patients

### 5.1. Primary Lung Cancer

In addition to hemoptysis, symptoms of lung cancer may include a new cough, shortness of breath, chest pain, unintended weight loss or recurrent respiratory infections. Pulmonary examination may demonstrate focal wheezing due to localized obstruction, reduced breath sounds with pleural effusion or lung mass, diffuse wheezing or a shift of the trachea in those with central airway obstruction [18,55]. More advanced findings include clubbing, cachexia or muscle weakness from a paraneoplastic syndrome.

Investigation of hemoptysis in those with suspicion of lung cancer typically starts with a chest radiograph. A chest radiograph could appear normal or reveal segmental atelectasis, lobar collapse, obstructive pneumonitis or pleural effusion. In those with an abnormal chest radiograph or significant risk factors (e.g., smokers, persistent hemoptysis, older), computed tomography (CT) of the chest with contrast and referral to a specialist is recommended [56]. A chest CT may show a range of abnormalities, from a solitary lung nodule to endobronchial obstruction with an atelectatic lobe or lung and/or pleural effusion, and contrast helps identify enlarged mediastinal/hilar lymphadenopathy and other vascular anomalies [18,55]. Bronchoscopy can help identify the source of bleeding, and, in cases with tumors, vascular lesions may obstruct airways distal to the tumor, and the mucosa may be friable or erythematous (Figure 1C,D). The tumor itself may be polypoid or papillary infiltrative with superficial erosions. When airway tumors outgrow their vascular supply, they tend to necrose, and deeper biopsies may be necessary in these cases [18,55].

Additionally, fluorodeoxyglucose (FDG)–positron emission tomography can the reveal positive uptake of FDG in metabolically active nodules and lymph nodes, indicating the presence of active disease. However, such uptake can arise from various conditions, including malignancy, active infection or inflammatory lung diseases. False-positive results can occur in infectious or inflammatory granulomas, while false negatives may occur in sub-solid nodules and carcinoid tumors [18,55]. Uptake within the mediastinal lymph nodes helps to guide the subsequent staging and diagnosis with endobronchial ultrasound-guided transbronchial needle aspiration for lung cancer [57].

### 5.2. Pulmonary Metastasis

Many cancers can metastasize to the lung, including melanoma, sarcomas and adenocarcinoma of the colon, breast, kidney or testicle [18,55]. Endobronchial lesions, in particular, can occur as metastases from renal cell carcinoma, thyroid carcinoma, esophageal cancer, ovarian cancer, melanoma, breast cancer, colorectal cancer and sarcomas. In addition to hemoptysis, symptoms from metastatic disease may include pain, weight loss, malaise, cough and dyspnea. A chest CT can reveal one or multiple nodules of various sizes, ranging from diffuse micronodular shadows (known as miliary) to well-defined masses, often irregular and located at the periphery of the lower lung zones. Some of these masses may also show signs of cavitation, and lymphadenopathy may be present [18,55]. Subsequent imaging to determine the primary site of the cancer may be required. Tissue diagnosis may be directed at other sites of disease or can be confirmed via bronchoscopic interventions.

## 6. Presentations and Investigations for Hemoptysis in Cancer Patients

There are limited data on the risk stratification of hemoptysis in cancer patients. A retrospective study by Grosu et al. that focused on solid organ malignancies found that advanced-stage diseases, active bleeding and endobronchial lesions during initial bronchoscopic evaluation were independently associated with decreased survival [58]. On the other hand, higher hemoglobin levels at the time of bronchoscopy and bleeding control at 48 h were linked to improved survival [58]. In hospitalized patients, mortality risk was increased with mechanical ventilation at referral, cancer diagnosis, aspergillosis, chronic alcoholism, pulmonary artery involvement and extensive lung infiltrates upon admission [3,18]. These predictors offer valuable insight into the prognosis of cancer patients with hemoptysis.

Mondoni et al. examined the long-term prognostic outcomes of patients with hemoptysis after 18 months of follow-up [59]. The overall mortality rate was 13.7%, with 43.4% of deaths happening in the first 3 months after presentation. Most deaths during the follow-up period were directly attributed to the underlying causes of hemoptysis, which were primarily pulmonary neoplasms. Indeed, malignancy emerged as the most frequent cause of hemoptysis in various studies, with notably higher mortality rates observed in some prior prospective studies of hemoptysis compared to Mondoni et al.’s findings [59]. Recent retrospective European studies have shown more diverse mortality rates from hemoptysis, with lung cancer remaining the primary cause of death [60,61,62]. Several independent risk factors for mortality in hemoptysis patients were advanced age, previous lung cancer diagnosis, smoking history and concurrent lung diseases [60].

In a study by Fartoukh et al., the severity of hemoptysis was assessed, and a scoring system was developed to predict in-hospital mortality. The overall in-hospital mortality rate was 6.5%, and independent predictors of mortality included chronic alcoholism, cancer as an underlying cause of the hemoptysis, involvement of the pulmonary artery as the source of bleeding, the involvement of two quadrants or more on chest X-ray at the time of referral and the initial use of mechanical ventilation [3]. The researchers created a prediction score based on these factors, which accurately estimated the probability of death in hemoptysis patients [3] and demonstrated comparable discriminative capabilities to those of the Simplified Acute Physiology Score II (SAPS II), a commonly used tool to assess acute disease severity. Categorizing patients into mortality risk groups based on the prediction score can enable physicians to plan appropriate treatment strategies, such as step-down facility management, vascular interventional radiology or ICU interventions [3].

Overall, the current literature sheds light on the long-term prognostic outcomes of patients with hemoptysis, emphasizing the significance of identifying the underlying cause, particularly malignancies, for predicting mortality. Additionally, these findings highlight the importance of developing a prediction score specific for cancer patients to assess the prognosis of patients with hemoptysis. The findings regarding mortality risk factors and prognosis in each study are summarized in Table 2.

## 7. Management Summary

### 7.1. General Scheme

**Figure 3:**
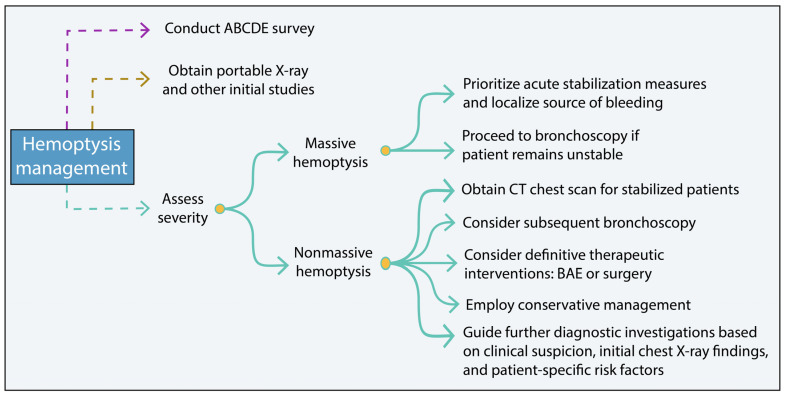
Hemoptysis management summary scheme. BAE, bronchial artery embolization.

When dealing with hemoptysis, it is crucial to consider even small amounts as potentially life-threatening until proven otherwise. The following steps should be adhered to (Figure 3):

1. Conduct an ABCDE survey to assess the patient’s overall condition and differentiate between hemoptysis (blood from the lower respiratory tract) and pseudohemoptysis (blood originating from the upper respiratory tract or gastrointestinal bleeding) [32,41].
2. Obtain a portable X-ray and other initial studies to aid in the evaluation of hemoptysis. These initial investigations help assess the severity of the hemoptysis, identify the underlying cause and guide appropriate treatment [32,41]. For minimal hemoptysis with a likely infectious etiology, antimicrobial therapy should be considered. In those with underlying risk factors, including immunosuppression, bronchiectasis or other structural lung disease, empiric antibiotics may also be helpful [68].
3. In cases of massive and/or life-threatening hemoptysis, prioritize acute stabilization measures and localize the source of bleeding based on the patient’s stability. If the patient remains persistently unstable, proceed directly to bronchoscopy to identify the bleeding source [69,70].
4. For stabilized/nonmassive cases, a CT chest scan should be obtained. Subsequent bronchoscopy may be considered for local thermoablative therapies, such as electrocautery, argon plasma coagulation, or Nd:YAG laser, especially for hemoptysis from malignant central airway disease [70]. In those cases not amenable to tracheobronchial intervention, definitive therapeutic interventions, such as bronchial artery embolization (BAE) or surgery may be warranted [69,70].
5. In cases of nonmassive hemoptysis, management is typically conservative. Clinical suspicion, initial chest X-ray findings, and patient-specific risk factors should guide further diagnostic investigations [69,70].

It is important to note that these steps serve as general guidelines, and the specific management approach may vary depending on the individual patient’s condition and clinical judgment [69,70].

### 7.2. Management of Hemoptysis in Cancer Patients

Control of hemoptysis often depends on the anatomic source, volume and rate of bleeding, clinical status and prognosis, and available resources (equipment, personnel and subspecialists, e.g., interventional pulmonologist, interventional radiologist and surgeons). Potential interventions can be bronchoscopic, endovascular via bronchial artery embolization, surgical or radiation. In bleeding due to a central airway source, tracheobronchial intervention may be feasible, but in bleeding due to a peripheral airway source, BAE is needed for acute bleeding control.

In patients whose hemoptysis is associated with lung cancer, mild hemoptysis typically does not require bronchoscopic procedures. In such cases of non-life-threatening hemoptysis, surgical resection is the optimal approach for operable lung tumors, while radiation therapy can be used when surgical options are not feasible.

**Figure 4:**
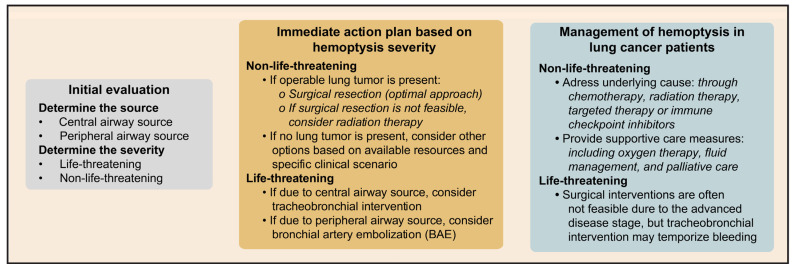
Hemoptysis management with considerations for lung cancer patients.

The prognosis for lung cancer patients with life-threatening hemoptysis is generally poor with high mortality rates. Surgical interventions are often not feasible due to the advanced disease stage, but tracheobronchial intervention may temporize bleeding. Treatment for nonmassive hemoptysis in lung cancer patients focuses on addressing the underlying cause through chemotherapy, radiation therapy, immune check point therapy or targeted therapy [24,25,71,72,73]. Supportive care measures, including oxygen therapy, fluid management and palliative care, are also important for symptom management and improving quality of life [74]. A multidisciplinary approach that considers the underlying cause, severity and overall health is necessary for effective hemoptysis management in lung cancer patients. Hemoptysis management is outlined in Figure 4.

#### 7.2.1. Initial Stabilization and Airway Preservation

In managing hemoptysis, appropriate measures should be taken to address acute bleeding. If necessary, a blood product transfusion should be initiated [75]. In cases of major hemoptysis, platelet transfusion to optimize the platelet count to 50,000 platelets/mm^3^ is recommended. In severe bleeding, reversal agents or antidotes to bleeding may include vitamin K, fresh frozen plasma, protamine, prothrombin complex concentrate or andexanet alfa and idarucizumab [76]. Prothrombin complex concentrate is utilized to manage severe coagulopathy or bleeding related to specific anticoagulant usage and/or vitamin K deficiency. This concentrate contains inactivated clotting factors, protein C, and protein S. The variant known as activated prothrombin complex concentrate is used when activated factors are required. Depending on the clinical scenario, replacement of specific coagulation factors, such as VIII, IX or XI, may be considered, particularly in individuals with known hemophilia. Also, adjunctive pharmacotherapy is crucial in the treatment strategy [75]. In those with DAH after allogeneic hematopoietic stem cell transplantation, systemic glucocorticoids are typically administered, but although aminocaproic acid is occasionally used, data for its benefit are lacking [77]. The use of recombinant human factor VII for refractory alveolar hemorrhage has been reported, but the risk of fatal thromboembolic events must be considered [78].

In treating hemoptysis, the first steps should be airway control, volume resuscitation, and treatment of any underlying bleeding disorders, along with monitoring in the ICU. Until airway control is achieved, patient posture can be used to reduce the aspiration of blood into the unaffected lung [79,80]. Putting the patient in the lateral decubitus position with the bleeding side down is recommended when the bleeding site is known. If the patient is able to expectorate secretions and protect their airway, then urgent palliative sedation or intubation is typically not necessary; however, if their work of breathing is not sustainable and/or not relieved with supportive measures, then emergent intubation with sedation may be warranted.

When precise airflow control to the affected lung is imperative, additional techniques for airway protection could be used. Single lung ventilation involves selectively ventilating one lung while isolating the other, which is essential for preventing blood from entering the healthy lung and maintaining oxygenation. This is typically achieved using a specialized endotracheal tube or bronchial blocker to block off one of the main bronchi, ensuring air reaches only the nonbleeding lung [81]. Double-lumen endotracheal tubes are another option, and they feature two lumens for separate lung ventilation; however, placement requires expertise, and lumen of the endotracheal tube may limit insertion of therapeutic bronchoscope [82]. Alternatively, in an emergent situation, the single lumen endotracheal tube can be advanced further into the nonbleeding lung, and the patient can be positioned with the bleeding side down. These techniques offer crucial airway control in hemoptysis management, preventing aspiration and protecting the unaffected lung.

Studies have shown that tranexamic acid, a synthetic antifibrinolytic drug, can be administered intravenously and via nebulizers to lessen hemoptysis and the need for interventional procedures in non-life-threatening hemoptysis cases. Tranexamic acid can be used as a stopgap therapy before the final intervention, even though its usefulness in treating life-threatening hemoptysis has not been properly examined [79,80].

In cases of life-threatening hemoptysis, intubation is frequently required for airway management. For endotracheal intubation, a single-lumen endotracheal tube with a diameter of 8.0 mm or more is frequently used, allowing for flexible bronchoscopy if necessary [4,69]. Flexible bronchoscopy can help eliminate blood from the airways so that breathing is adequate. Cold saline lavage, topical application of vasoconstrictive medications (such as epinephrine) and bronchial blockers are therapeutic methods used through the bronchoscope to achieve hemostasis. Regional thermally ablative procedures, such as electrocautery and application of argon plasma coagulation, alongside Nd:YAG laser, can also yield hemostatic effects [70]. When at hand, inflexible bronchoscopy affords heightened airway steadiness by enabling the prompt and discriminative isolation of individual mainstem bronchi, all the while ensuring ventilation. This approach is frequently synergized with adaptable bronchoscopy. The broader lumen of the rigid endoscope enables the simultaneous utilization of specialized implements, encompassing bronchial blockers, thermal ablation fibers, cautery apparatus or instruments for tissue excision and hemostatic tamponade.

#### 7.2.2. Endovascular Intervention

Hemoptysis can be diagnosed and treated by angiography and embolization. BAE is the main strategy for handling cases that pose a threat to life. However, localizing the bleeding location can be difficult and necessitates preprocedural imaging methods, such as chest CT and bronchoscopy. Interventional radiologists use diagnostic angiography to locate the bleeding vessel. They check for swollen or distorted arteries, contrast extravasation and hypervascularity. During super-selective artery embolization, microparticle embolic agents like polyvinyl alcohol (PVA) particles or gelatin microspheres are utilized. The nonbronchial systemic and pulmonary circulations are assessed if the bleeding source cannot be localized to the bronchial circulation. The control of hemoptysis can be improved by embolizing nonbronchial systemic collaterals during BAE, and pulmonary artery angiography can identify anomalies such as a Rasmussen aneurysm [69,83,84,85]. The most dreaded complication of BAE is spinal cord ischemia secondary to inadvertent embolization of anterior or posterior spinal arteries, and the risk ranges from 1.4% to 6.5% in published series [86].

BAE in cancer patients has distinct outcomes. Although immediate control of bleeding can be achieved, recurrence of bleeding may occur in 20% to 53% of cancer patients; and possible explanations for this rebleeding include progression of underlying disease, recanalization or revascularization, incomplete embolization and emergence of other blood supply to the affected area (such as from systemic collaterals) [87]. In a study of lung cancer patients, Han and associates noted that massive hemoptysis and cavitary lung mass were significant predictors of shortened hemoptysis-free survival [88]. Interestingly, in a small study from our institution, Wang and colleagues found that among those with hemoptysis not related to malignant disease in the lung, survival after BAE was significantly better compared to those with tumor-related hemoptysis [89].

#### 7.2.3. Bronchial Blocking Techniques

Bronchial blockade is a crucial strategy for managing massive hemoptysis, serving two main purposes. Firstly, it prevents blood from entering the healthy lung, avoiding contamination and facilitating continued ventilation of the unaffected side. Secondly, it offers stability, allowing time for further interventions like interventional radiology or surgery [29,90]. Occasionally, bronchial blockade can induce clot formation and temporary hemostasis. A bronchial blocker, like the Arndt blocker or Cohen Flexitip blocker, is employed during surgical procedures requiring single-lung ventilation to isolate the bleeding lung while maintaining ventilation in the remaining lung. This flexible catheter is guided through the endotracheal tube and placed with bronchoscopic guidance. The cuff at its distal end can be inflated in the mainstem or lobar branches. Familiarity with troubleshooting and monitoring of bronchial blocker position is paramount, as dislodgement of the bronchial blocker can result in ventilatory compromise. Once hemostasis is achieved, the balloon is deflated, and the blocker is removed to prevent complications like atelectasis and pneumonia [29,91,92,93,94]. Balloon tamponade can also be achieved using Fogarty catheters but cannot be left in the airway [29,95]. Another option is using a pulmonary artery (PA) catheter, allowing for selective deployment into smaller bronchi while maintaining ventilation. This technique has been successful in some cases but is not routine [29,96,97].

#### 7.2.4. Role of Surgery

With advances in interventional radiology, surgical interventions for massive hemoptysis typically follow first-line nonsurgical approaches to control bleeding [98]. Lung resection in the form of lobectomy can be a life-saving procedure, but it must be performed selectively to optimize the operative conditions and improve patient outcomes [99]. In a series of 813 patients with severe hemoptysis, as reported by Andrejak et al., 111 patients underwent surgical resection; among these surgical patients, interventional radiology intervention was attempted in 78%, surgery was performed emergently in 43%, scheduled after bleeding control in 43% and planned after discharge in 14% [98]. The main indications for surgery in this series included mycetoma, cancer, bronchiectasis and active tuberculosis.

Studies have revealed that emergency lung resection procedures have much greater fatality rates than elective ones. Current guidelines reserve immediate surgical intervention for certain circumstances, such as acute chest injury, iatrogenic pulmonary artery rupture or pulmonary artery hemorrhage combined with a resectable lung tumor [83,98,100]. A definitive surgical procedure must be carefully considered for patients with high-risk rebleeding following BAE. Lower mortality rates and surgical morbidity have been attained due to a delay of initial surgical intervention in favor of BAE [83,98,100]. In instances where hemoptysis is attributed to mycetoma, surgical resection may also be considered a viable option. This applies to both small, localized mycetoma lesions and extensive lesions, as surgical resection reduces the mycetoma burden and augments the effectiveness of medical treatment. Post-operative sequelae in pulmonary resection for massive hemoptysis in nonmalignant conditions may results in complications in 25% of cases, including thoracic hemorrhage, bronchopleural fistula, air leak, pulmonary embolism, atelectasis and lung infection [101]. Thus, surgery for massive hemoptysis may engender significant morbidity and mortality, and it is reserved for select cases and scenarios with multidisciplinary consensus.

## 8. Conclusions

Hemoptysis, which can range from mild to severe cases, is often caused by various factors, including lung cancer, where it occurs in approximately 20% of cases. Accurately assessing the clinical impact and implications of bleeding is crucial because of the potential unreliability of volume-based definitions. The effective treatment of hemoptysis involves addressing the underlying cause. In cases of major bleeding, immediate management may require transfusion, bronchoscopic intervention or bronchial artery embolization. Further development of a specific prediction score for hemoptysis in cancer patients will be vital to stratify cases of hemoptysis and guide interventions to stabilize, direct observation or hospital admission and prognosticate outcomes. Implementing such a score would optimize resource allocation, enable timely interventions and improve patient outcomes by effectively addressing the root cause and managing episodes of acute bleeding.

## Figures and Tables

**Table 1:** Etiology of hemoptysis in general and specific to cancer patients [21,29,30,31].

Category	Disease
**Pulmonary**	Neoplasm *, e.g., bronchogenic carcinoma (second most common cause) Broncho-vascular fistula resulting from bronchopleural fistula [32], causing massive hemoptysis Tumors infiltrating the bronchial wall [33] Transformation of adenocarcinoma into squamous cell carcinoma [34] Endobronchial tumors (may present with hemoptysis in 15% of patients) Infection Tuberculosis (most common cause worldwide) Acute viral or bacterial infection Mucormycosis [35] Staphylococcus aureus [36] Invasive aspergillosis [35] Aspergillus mycetoma (fungus ball) Strongyloidiasis [37] Nontuberculous mycobacterial (Ntm) [38] Coccidioidomycosis [39] Actinomycosis [40] Pyogenic lung abscess [41] Cystic fibrosis Bronchiectasis * Lupus pneumonitis due to systemic lupus erythematosus
**Cardiac**	Mitral stenosis * Congestive heart failure * Pulmonary hypertension Congenital heart disease *
**Vascular**	Pulmonary embolism Vasculitis Goodpasture syndrome Behcet disease Granulomatosis with polyangiitis Pulmonary vascular fistula Vascular malformation (e.g., pulmonary artery aneurysm, arteriovenous malformations) * Pulmonary artery rupture Tracheoinnominate artery fistula Hereditary hemorrhagic telangiectasia
DAH * [42,43,44,45]	Typically presents with dyspnea, cough, hemoptysis and new alveolar infiltrates on chest imaging Hemoptysis is common but may be absent in up to one-third of patients
**Hematologic**	Anticoagulant use Coagulopathy Thrombocytopenia
**Trauma**	Airway trauma Lung contusion Foreign body
**Iatrogenic**	Injury to structures * during: Pulmonary artery catheterization Lung biopsy Airway stenting Right heart catheterization
**Drugs and toxins**	Penicillamine [46,47] Crack cocaine [22,48] Bevacizumab [13]
**Other**	Thoracic endometriosis (catamenial hemoptysis) Cryptogenic: no identified cause on CT or bronchoscopy (50% of cases in high-income countries) Idiopathic pulmonary hemosiderosis

* Causes of life-threating hemoptysis.

**Table 2:** Summary of the risk factors and prognosis in the published literature.

Study/Author(s)	Cohort	Risk Factors for Mortality, Prognosis, Other Findings
**Grosu et al.** [58]	Retrospective Patients with solid organ tumors and mild hemoptysis N = 112	Upon multivariate analysis, factors independently associated with improved survival had higher hemoglobin values (HR 0.78; 95% CI, 0.67–0.91) and cessation of hemoptysis without recurrence at 48 h (HR 0.43; 95% CI 0.22–0.84). Variables independently associated with worse survival were disease stage (HR 10.8; 95% CI, 2.53–46.08) and active bleeding with endobronchial lesion (HR 3.20; 95% CI 1.74–5.89).
**Fartoukh et al.** [3]	Retrospective Consecutive patients admitted to ICU with hemoptysis N = 1087	Independent predictors of mortality were mechanical ventilation at the time of referral, cancer diagnosis, aspergillosis, chronic alcoholism, pulmonary artery involvement and infiltrates involving two or more quadrants upon admission. A model-based score for prognosis was developed that assigned 1 point for chronic alcoholism, pulmonary artery involvement and radiographic patterns and 2 points for cancer, aspergillosis and mechanical ventilation.
**Hirshberg et al., Vanni et al., Soares et al., and Uzun et al.** [6,62,63,64]	Analytical cohort studies	Malignancy was a leading cause of hemoptysis, with a decrease in mortality related to bronchiectasis, lower respiratory tract infections and other less frequent causes.
**Uzun et al. and Tsoumakidou et al.** [64,65]	Analytical cohort studies	Malignancy was a leading underlying cause of hemoptysis with mortality rates ranging from 19.5% to 22%.
**Soares et al., Petersen et al., and Abdulmalak et al.** [60,61,62]	Analytical cohort studies	Lung cancer was the primary cause. Reported mortality rates varied significantly, ranging from 5.9% to 27%.
**Petersen et al.** [60]	Retrospective Consecutive patients with no malignancy suspected on chest CT N = 609	Predictors of mortality were advanced age, a previous lung cancer diagnosis, a current or previous smoking history, and concurrent lung diseases.
**Mondoni et al.** [59,66]	2019 study: secondary analysis of an observational multicenter study N = 486 2021 study: prospective multicenter study N = 606	Recurrences indicated previously undetected pathological findings, as there was a recurrence of hemoptysis in 7 patients, of whom 3 were found to have lung cancer upon further investigation. Pulmonary neoplasms were the primary cause of death, and the overall mortality rate was 13.7%.
**Tsoumakidou et al.** [65]	Prospective cohort N = 184	No patients initially diagnosed with an etiology other than lung cancer were found to have lung cancer upon further investigation.
**Abdulmalak et al.** [61]	A 5-year retrospective cohort study N = 81,572	An initial diagnosis of respiratory infection with highest lung cancer detection rate (10.4%) during the follow-up, and lung cancer was the cause in 17.4% of patients.
**Majhail et al.** [67]	Prospective data review of patients who had hematopoietic stem cell transplantation (HSCT) with alveolar hemorrhage N = 116	Advanced age, utilization of an allogeneic donor source, administration of a myeloablative conditioning regimen and the occurrence of acute severe graft-versus-host-disease were identified as independent predictors associated with a heightened risk of alveolar hemorrhage following HSCT. The probability of 60-day survival from the onset of hemorrhage was determined to be 16% in the diffuse alveolar hemorrhage group and 32% for the idiopathic alveolar hemorrhage group. With the exception of 20 patients, all individuals in this study received a standard regimen of high-dose corticosteroids; among the patients who received corticosteroids, the 60-day survival rate was found to be 26%, while those who did not receive corticosteroids exhibited a 60-day survival rate of 25%.

HR, hazard ratio; CI, confidence interval; CT, computed tomography.

## Data Availability

All data collected in this study are provided in this paper.
